# Community pharmacists’ attitudes toward the quality and price of locally manufactured generic medicines in Kabul, Afghanistan

**DOI:** 10.1186/s40545-015-0037-3

**Published:** 2015-05-18

**Authors:** Mohammad Bashaar, Mohamed Azmi Hassali, Fahad Saleem

**Affiliations:** Discipline of Social and Administrative Pharmacy, School of Pharmaceutical Sciences, Universiti Sains Malaysia, 11800 Pulau Pinang, Malaysia

**Keywords:** Afghanistan, Community pharmacist, Generic medicine, Price, Quality

## Abstract

**Objective:**

To report the attitudes of community pharmacists in Kabul, Afghanistan, concerning the quality and price of locally manufactured medicines.

**Methods:**

A cross-sectional descriptive study, involving a sample of 198 community pharmacists was conducted in Kabul city.

**Results:**

With a response rate of 100%, most of the respondents 70.7% had 11–20 years experience working as a pharmacist. About 84.3% of the pharmacists dispensed imported generic medicines from Pakistan, Iran, India, and the UAE. Only 15.7% of pharmacists were dispensing locally produced generics from Ariana (i.e. a local pharmaceutical manufacturer). Exactly half of the pharmacists 50.0% reported that locally produced generics were equally safe and efficacious as the imported generics, while 70.7% of the respondents believed that the local manufacturers of generic products had reliable logistics and supply systems. However, 80.8% of respondents expressed concerns regarding their own credibility when stocking the medicines. Consequently, 80.3% of the sample only stocked well-advertised domestic generics, which were likely to be seen by consumers as more credible alternatives. Most of the respondents 82.8% were confident that the locally manufactured generics were cheaper than imported generics. Interestingly, 80.8% of the respondents favoured the establishment of a national brand substitution policy. Furthermore, 90.4% of the respondents believed that it was the responsibility of the Afghan regulatory authorities to educate pharmacists on the quality of domestic generics.

**Conclusion:**

Although community pharmacists had a positive attitude towards the quality and affordability of locally manufactured medicines, due to lack of resources most of their medicines are imported. Despite their positivity towards the quality and price of generics, the community pharmacists only dispense them to a minimal degree, because of low local production levels among other reasons. The findings call for improvements in the local pharmaceutical industry in order to substitute imported medicines with local generics. The government must take firm steps to formulate and reinforce pharmaceutical pricing and brand substitution policies to help in controlling healthcare costs. Further research, especially a countrywide survey, is required.

## Introduction

Prior to 1979, Afghanistan had sufficient domestic pharmaceutical manufacturing to meet local demand, as well as export capacity [1,2]. Due to decades of conflict, the pharmaceutical industry collapsed and remained “understudied” [3], being one of the least developed industrial sectors in the world, functioning with many weaknesses and in a disorganised manner [4]. According to WHO, the overall pharmaceutical situation in Afghanistan has deteriorated because health infrastructure, machinery, laboratories and buildings have been seriously damaged or destroyed [5].

However, despite these deficiencies, after 2001 a few multinational pharmaceutical companies started importing medicines to Afghanistan and 14 small and medium-sized privately owned pharmaceutical manufacturing companies have contributed to the growth of the industry [6]. Nonetheless, according to a recent assessment of domestic pharmaceutical manufacturing capacity in Afghanistan, the sector is facing a number of critical issues [1]. For example, no licensing authority that issues Good Distribution Practices (GDP) licenses exists and there are no national guidelines on GDP [2]. The medicine supply system is critically under-regulated with weak provincial management leading to an erratic supply situation [4]. There is no national Good Manufacturing Practice (GMP) or Adverse Drug Reaction (ADR) guidelines, and a medication error reporting system is lacking. In addition a pharmacovigilance system has not yet been introduced [7]. Due to a lack of sufficient budget, human resources and infrastructure, the implementation of policies, legislation, and regulations is slow.

To address these outstanding issues in the pharmaceutical sector, the Ministry of Public Health (MOPH) has developed a strategic plan for 2011–2015 [8]. One of the objectives of this strategic plan is to establish and implement a health governance system to ensure transparency and efficiency in pharmaceutical supply chain management, and increase local pharmaceutical production capacity [8]. Under this objective, the government has shown its commitment to improve the production of locally manufactured generic medicines. They have recognised that soaring medicine costs could be controlled by promoting the use of affordable, safe and effective generic medicines instead of their more expensive branded equivalents [9,10].

A generic medicine is a pharmaceutical product, usually intended to be interchangeable with an innovator product, that is manufactured without a license from the innovator company, and is marketed after the patent or any other exclusive rights belonging to the innovator company have expired [11]. Stringent quality measures must be undertaken and the generic medicine must meet the standards of the originator brand in order to be considered safe [12,13].

Due to lower prices, generic medicines can yield significant healthcare cost savings and these savings can be used to subsidise expensive novel medicines and support pharmaceutical research and development [14]. The first low-cost generic medicine program in the United States, introduced in September 2006 by Wal-Mart, offered a limited number of generic medicines at a cost of US$4 for a month’s supply of each drug [15]. Similarly, in the UK, the government has provided strong incentives to promote generic prescription and use [14]. Many other governments have also advocated shifting to lower cost generic medications in order to improve affordability, reduce healthcare spending, and promote better adherence to drug regimens [16-18]. In France, controlling pharmaceutical expenditure has been a policy priority for many years and policies favouring generic medications have featured prominently [19]. Direct price control policies are a common phenomenon, even in generic medicine markets, France being just one example. The French authorities stipulate that the price of a generic medicine must be 30% lower than that of equivalent branded medicines [20]. Similarly, the Danish medicines agency introduced “Medicine Profile,” a database for both general practitioners and patients to check their medicines and to compare the price of innovator medicines with generic alternatives [21]. The Danish generic medicine policy gave rise to conditions favouring the development of a low-price high-volume generic medicine market [21]. In Portugal, pro-generic medicine media campaigns were undertaken resulting in increased demand [21].

Where a favourable market for generic medicines has been developed, health care has become significantly more affordable and its universal coverage more sustainable [21]. Generic medicines allow governments to exercise greater fiscal control over their pharmaceutical expenditure because generic medicines should ideally be as safe, and of the same quality and therapeutic efficacy, as their branded equivalent medicines [21]. Therefore, efforts should be made to extend generic medicine public awareness programs and advocacy activities to include more detailed information, so that patients have a greater understanding of brand substitution practices. It is equally critical that physicians and pharmacists have an accurate understanding of brand substitution, and that they provide accurate information to patients [22].

The affordability of prescription medicines is one of the most pressing public health issues in the United States. According to at least one study, many patients self-administer less than their prescribed dose so as to make medications last longer, or fail to fill prescriptions because of the cost [15]. Consequently, brand substitution is supported by health authorities in many countries throughout the world because it provides cheaper alternatives to innovator medicines [23]. However, according to our knowledge, no brand substitution or generic medicine prescribing policies exists in Afghanistan and there is a lack of research in this area. Pharmacists play a pivotal role and as healthcare providers, therefore it is imperative that they know about the price and quality of generic medicines. Additionally, it is crucial that policy makers understand these matters in order to establish a sound generic medicine policy leading to substantial savings in health care expenditure and improving access to essential medicines in the country [24,25].

Therefore, in this study, we aimed to explore the attitudes of community pharmacists in Kabul regarding the quality and price of locally manufactured generic medicines.

## Methods

This cross-sectional study relied on a convenience sample of community pharmacists from Kabul, Afghanistan. Prior to conducting the study, ethical approval was granted by the local Institutional Review Board (IRB). Over a four-month period, from March–June 2013, respondents were approached and asked to participate in the study. The questionnaire had previously been used in Penang, Malaysia [26], and approval for use of the questionnaire in the present study was granted by the author. One question was added in order to assess the community pharmacists’ attitudes regarding the affordability of domestic generic medicines. The draft questionnaire was reviewed for accuracy and validity, then translated and back-translated into Dari (Farsi). Minor revisions were made to the translated questionnaire after initial pilot testing.

The final questionnaire that was administered to participants was in three sections. The first section collected participants’ demographic information with the help of five questions. The second section inquired about the pharmaceutical companies (Abbot, GSK, Getz, Julphar, Exir, Gracure and Ariana), from which the pharmacists were sourcing their generic medicine stocks. The six international companies and one local company (Ariana) were included because of their popularity, medicine stocks and the retailers’ inclination towards procurement from them. From the list of seven companies, the respondents were asked to identity from which they obtained their stocks. In the third section, eleven questions asked about the community pharmacists’ views on issues concerning locally manufactured generic medicines. In this section of the questionnaire the respondents were asked to indicate their responses on a five-point Likert scale (1 = strongly agree, 2 = agree, 3 = neutral, 4 = disagree, 5 = strongly disagree).

A total of 198 community pharmacists were approached for participation in this study by visiting a range of public and private hospitals, clinics, and pharmacies (i.e. medical outlets) across Kabul. Participation was voluntary and the anonymity of the respondents was preserved. There was no part of the questionnaire for the recording of identifying personal information. After the completed questionnaires were collected, the data was analysed with SPSS 16.0 for descriptive analysis, using frequency analysis and cross-tabulation to examine the data characteristics and to summarise the overall results.

## Results

### Demographic information of the respondents

The basic demographic information of the respondents is summarised in Table 1. All of the 198 participants took part in the survey, where 70.7% had 11–20 years of pharmacy practice in the community. The majority of respondents 99% were male and 87.3% of the respondents were aged 30–50 years. Experienced community pharmacists were more inclined to know about generic medicine and dispense it. Among all the interviewees, 94.9% had graduated from local universities.

**Table 1:** **Respondent demographics**

***Demographic Information***		***Frequency (%)***	
***Gender***	Male	196	(99.0)
	Female	2	(1.0)
***Age***	Under 30	15	(7.6)
	30–40	46	(23.2)
	41–50	127	(64.1)
	Over 50	10	(5)
***Year of Graduation***	Before 1980	4	(2)
	1981–1990	36	(18.2)
	1991–2000	135	(68.2)
	After 2001	23	(11.6)
***Years of practice in community pharmacy***	Less than 10	44	(22.2)
	11–20	140	(70.7)
	21–30	14	(7.1)
***Country of Graduation***	Afghanistan	188	(94.9)
	Foreign	10	(5.1)

### Findings on the stocking and dispensing of generic medicines

When assessing the community pharmacists’ generic medicine stocking and dispensing behaviour, it was found that the majority of respondents 84.3% were dispensing imported generic medicines from Pakistan, Iran, India, and the UAE (Table 2). Ariana, one of several local Afghan generic pharmaceutical companies, was used by only 15.7% of pharmacists. Nearly half 45.9% of the imported generic medicines dispensed by pharmacists were from Abbot 10.1%, GSK 14.1%, and Getz (a Pakistani pharmaceutical manufacturer, 21.7%. Exir (an Iranian pharmaceutical company) was selected by 23.2% of the pharmacists, showing that this company has a good market in Kabul.

**Table 2:** **Pharmaceutical companies where pharmacists purchase their generic medicine**

**Pharmaceutical Company**	**Country of Origin**	**Frequency**	**%**
Abbot	Pakistan	20	(10.1)
Julphar	UAE	18	(9.1)
GSK	Pakistan	28	(14.1)
Exir	Iran	46	(23.2)
Getz	Pakistan	43	(21.7)
Gracure	India	12	(6.1)
Ariana	Afghanistan	31	(15.7)

### Community pharmacists’ attitudes towards the quality and price of locally manufactured generic medicines

When respondents were asked about their views of the quality and price of locally produced generic medicines, 50.0% indicated that the locally produced generics were as safe and efficacious as imported generics. Furthermore, 70.7% of the respondents believed that local generic manufacturers had reliable logistics and supply systems. Notwithstanding, only half of the respondents 50.0% preferred to stock locally manufactured generics even though the local manufacturers provided better bonus schemes than the pharmaceutical importers. The majority of respondents 82.8% also indicated having concerns about the credibility of manufacturers/suppliers when stocking generic medicines. In addition, most of the respondents 80.3% reported that they would only stock locally manufactured generics that had been well advertised in both the general and medical/professional media (Table 3).

**Table 3:** **Community pharmacists’ attitude towards the quality and prices of locally manufactured generic medicines**

**Question**	**Survey questions/Statement**	**Frequency (%)**										
		**1**		**2**		**3**		**4**		**5**		**No answer**
1	Locally manufactured generics are equal in their quality compared to the imported generics.	71	(35.9)	23	(11.6)		(0)	78	(39.4)	22	(11.1)	4 (2.0)
2	Locally manufactured generics are equal in their safety and efficacy compared to the imported generic.	24	(12.1)	75	(37.9)	4	(2)	83	(41.9)	8	(4)	4 (2.0)
3	Manufacturers of local generic products have a reliable logistic and supply system.	56	(28.3)	84	(42.4)	16	(8.1)	28	(14.1)	9	(4.5)	5 (2.5)
4	I prefer to stock and dispense locally manufactured generics because the companies provide good bonus scheme compared to suppliers importing them.	46	(23.2)	53	(26.8)	6	(3)	77	(38.9)	11	(5.6)	5 (2.5)
5	Credibility of the generic manufactures/suppliers are my concern when stocking medicines in my pharmacy.	103	(52)	61	(30.8)	3	(1.5)	23	(11.6)	2	(1)	6 (3.0)
6	I will only stock locally manufactured product which is well advertised through medical representatives and medicine related references.	117	(59.1)	42	(21.2)	6	(3)	20	(10.1)	8	(4)	5 (2.5)
7	Imported generics need to pass more stringent approval process compared with locally manufactured ones.	98	(49.5)	75	(37.9)	9	(4.5)	9	(4.5)	3	(1.5)	4 (2.0)
8	Locally manufactured generics are cheaper compared to imported generics.	121	(61.1)	43	(21.7)	5	(2.5)	19	(9.6)	6	(3)	4 (2.0)
9	Locally manufactured generics are affordable to be purchased in comparison to imported generics.	55	(27.8)	60	(30.3)	6	(3)	21	(10.6)	51	(25.8)	5 (2.5)
10	I believe we need to establish brand substitution policy in Afghanistan.	132	(66.7)	28	(14.1)	7	(3.5)	16	(8.1)	10	(5.1)	5 (2.5)
11	The Afghan Drug Regulatory Authorities need to convince pharmacists that registered locally manufactured generics are of high quality and standards.	133	(67.2)	46	(23.2)	8	(4)	4	(2)	2	(1)	5 (2.5)

In the opinion of most of the respondents 87.4% imported generics are required to pass through more stringent approval processes, as their origin is not known. Also, the majority of respondents 82.8% agreed that the locally manufactured generics were cheaper than the imported generics. Just over half 58.1% of the respondents believed that local generics are more affordable than imported generics. Interestingly, 80.8% of the respondents agreed that Afghanistan should establish a national brand substitution policy and further, the majority of the respondents 90.4% indicated that the Afghan drug regulatory authorities need to convince pharmacists that registered locally manufactured generics are of high quality and standards.

## Discussion

In Afghanistan, as in many other low-income post-conflict settings, there are more imported medicines than locally produced medicines and hence there are significant concerns regarding quality and price. The Afghan pharmaceutical market is largely dependent on privately imported medicines due to a lack of local pharmaceutical companies and infrastructure. Afghanistan imports over 95% of its medicines from other countries [27]. In the current study, we found that majority of the respondents chose to stock imported generic medicines (Table 2); therefore, our findings are consistent with those of Paterson [6].

Low-income countries are commonly believed to have poor quality health services [28] and Afghanistan is no exception. Due to three decades of conflict, the situation has deteriorated. The Afghan pharmaceutical and health care system, including the supply of medicines, remains largely ad hoc and disorganised, with little production capacity of its own. Many actors, including government ministries, non-governmental organisations, foreign mission hospitals, and the private sector make up the system. Each of these actors is largely independent of the others and is responsible for their own procurement, logistics, supplies, and distribution networks [4,6]. The General Directorate for Pharmaceutical Affairs (GDPA), in addition to regulating the local pharmaceutical industry, is charged with the responsibility of ensuring the safety and quality of imported medicines [2]. However, for multiple reasons, including a lack of funding, limited human resources, and a lack of clear policy direction, the GDPA is sorely limited in its capacity to execute this monitoring role.

As mentioned earlier, more than half of the respondents felt that locally produced generics were as safe and as efficacious as imported generic medicines. This finding is consistent with international studies, which also find that local generics are perceived to be similarly safe and effective compared to imported generics [26]. However, the quality of imported medicines is often questioned by the public, with low quality, spurious, counterfeit, and expired medicines entering the country through legal loopholes or otherwise smuggled into the country. Furthermore, much of the patient population is illiterate and naive about pharmaceutical manufacturing and expiry dates. Therefore, to maintain the trust of both community pharmacists and patients alike, the Afghan regulatory authorities should implement more stringent regulation, including higher standards of quality management, guaranteeing the quality of imported generic medicines by performing quality control laboratory exams, and ensure good distribution practices. This argument is not without precedence, a Malaysian study similarly finding support for imported generics needing to pass a more stringent approval processes [26]. However, in many post-conflict settings, government regulations exist only on paper or serve only to benefit corrupt or ineffective government officials [29].

The medicines available in Afghanistan tend to be of poor quality. This is partially attributable to the scarcity of resources available to the National Medicines and Food Board (NMFB) to properly control and assess the quality of medicines. Another reason underlying the poor quality of available medicines concerns the role of importers and distributors. While every effort is made to import medicines which should be cheap and affordable, as with any private enterprise it is imperative to make a profit, and handsome profits can be made by subverting the regulatory system [30]. Consequently, alternative and black market supply channels allow for the introduction of expired and otherwise cheaper poor quality generics to enter the market. In addition, the practice of counterfeiting medications is not unheard of in Afghanistan. Therefore, the use of local generic medicines can be enhanced by improving their quality, instituting appropriate brand substitution policies, and by educating consumers [29,31]. This is clearly essential, since the economic goal of cost containment by generic substitution is worthless if it causes death instead of cure [32].

In the current study, most of the respondents recommended that the Afghan drug regulatory authorities should be working to convince pharmacists that registered, locally manufactured generics are of as high a quality as most imported medicines and are manufactured in compliance with international standards. In addition, more than half of the participating pharmacists agreed that domestic generics are cheaper than imported generics. Similarly, the Malaysian study also found that domestic generics were cheaper than their imported counterparts [26]. Imported medicines tend to be more expensive due to various taxes, tariffs, and other duties along the procurement chain. Brand substitution is an effective cost-containment solution to this problem.

Healthcare authorities throughout the world encourage the practice of generic substitutions [33], and Afghanistan is no exception. Therefore, in this study, most of the respondents strongly supported the establishment of a national brand substitution policy in Afghanistan. These findings are almost identical to those of Hassali et al. [26].

Brand substitution has become common practice in the United States since the late 1970s [13]. Widespread brand substitution of outpatient prescription drugs has saved approximately US$8.8 billion, or approximately 11% of the pharmaceutical budget for adults, in the United States each year [34,35]. Similarly, brand substitution by pharmacists is standard practice in UK hospitals and is being proposed for implementation in primary care [13]. Although 83% of community or primary care physicians in the UK already write prescriptions for generic medicines [25], substituting more expensive generic brands with even less expensive generic brands might still result in further savings. Therefore, efforts to promote brand substitution should be targeted first and foremost at the point of prescription, the treating physician [25]. As the earlier Malaysian study revealed, the desire to maintain a high profit margin is one of the main reasons that community pharmacists practice brand substitution [29,36].

Brand substitution is a collaborative act, therefore the pharmacists and patients must be informed and trained [36] since, at the dispensing point, the pharmacist plays an important role in helping patients choose between branded or generic medicines [15,23,25]. Therefore, the undergraduate pharmacy and medical curricula may need to be updated and adapted especially in the regulatory and quality control aspects of generic medicines, and further bioequivalency requirements must be applied to the registration and production of generic medicines [26,37].

From this study, it is clear that community pharmacists in Kabul city have a positive attitude towards the quality and price of locally manufactured medicines, the implementation of generic substitution policy, and the application of stringent quality control systems. This positive attitude is an opportunity for the health authorities to embark on development and reinforcement of generic medicine policy in Afghanistan. This can lead to improved access to quality assured locally manufactured medicine and savings on healthcare costs both for patient and government.

### Limitations of the study

The study has the limitations inherent in most survey-type studies, for example a hardcopy questionnaire was used for data collection, meaning there is a chance that subjects refer to the internet or other educational resources to answer the knowledge-based questions [38]. Further, there is a possibility of a social desirability response bias on the attitude issues, where the interviewees might be inclined to provide more favourable responses toward generic medicine [38,39]. Finally, this study was conducted in Kabul city focusing on community pharmacists; therefore, the sample and results may not be representative of the attitudes of all community pharmacists and all cities in the country.

## Conclusion

The findings of the current study indicate a positive attitude among community pharmacists towards locally manufactured medicines. Although they appeared to believe that quality generic medicines are affordable and would result in significant cost savings, due to lack of pharmaceutical manufacturing resources most medicines are imported. Additionally, the community pharmacists generally expressed positive attitudes towards the quality and price of locally manufactured generics, but dispense them in minimal amounts, due to a low level of local production. This suggests that domestic pharmaceutical companies should be empowered to produce substitutes for imported medicines. Further, now may be the ideal time for the government to embark on a process of formulating explicit pharmaceutical pricing and brand substitution policies in order to exercise greater control over health care costs.
